# Views of cognitive aging in midlife and older age: development of a new scale

**DOI:** 10.3389/fpsyg.2026.1837995

**Published:** 2026-05-20

**Authors:** Enrico Sella, Elena Carbone, Paolo Ghisletta, Erika Borella

**Affiliations:** 1 Department of General Psychology, University of Padova, Padova, Italy; 2 Department of Psychology, Faculty of Psychology and Educational Sciences, University of Geneva, Geneva, Switzerland

**Keywords:** cognition, measurement development, subjective aging, views of aging, views of cognitive aging

## Abstract

**Introduction:**

Perceptions, beliefs, expectations, and attitudes about aging and one's own aging (i.e., subjective views of aging [VoA]) play a crucial role in shaping individuals' health and well-being outcomes, including cognitive functioning in older adulthood. However, no existing questionnaire specifically assesses VoA focusing on perceptions and beliefs about age-related cognitive changes with aging. We aimed to develop and validate the Views of Cognitive Aging (VoCA) questionnaire, comprising two sections assessing generalized (VoCA-G) and personal (VoCA-P) views of cognitive aging, and to examine its associations with VoA measures and cognitive functioning.

**Methods:**

A total of 727 participants (50–84 years old) completed the VoCA, assessing generalized and personal views of cognitive aging. They also completed generalized ([Non] Essentialist Beliefs about Aging) and personal VoA (felt age, Attitudes Toward Own Aging, Awareness of Age-Related Change, and Aging Perceptions Questionnaire) measures. Attention, working memory, and processing speed tasks were administered to examine cognitive functioning.

**Results:**

For each VoCA section, factor analyses showed a two-factor structure representing nonessentialist (-nE; malleable) views (VoCA-GnE, VoCA-PnE) and essentialist (-E; fixed) views (VoCA-GE, VoCA-PE). The VoCA showed good reliability and validity with VoA measures. Moreover, VoCA-GnE and VoCA-PnE were positively correlated with working memory, attention, and processing speed whereas VoCA-GE and VoCA-PE were negatively correlated with these cognitive measures.

**Conclusions:**

The VoCA, a novel reliable instrument, assesses generalized and personal essentialist and nonessentialist views about cognitive aging. It enables capturing middle-aged and older adults' VoA related to cognitive changes and supporting identification of those at risk for cognitive complaints as well as guiding interventions to promote successful aging.

## 1 Introduction

Subjective views of aging (VoA) represent a broad construct referring to individuals' beliefs, experiences, and evaluations of older adults as a social group and of their own aging process (Shrira et al., 2022). It includes generalized VoA, that is, socially shared beliefs about older adults and the aging process in general, and personal VoA, that is, individuals' representations regarding their own aging process and the state of being old (Diehl et al., 2014; Shrira et al., 2022). Old age stereotypes; attitudes toward aging; and the more recent concept of essentialist and nonessentialist beliefs about aging, capturing individuals' VoA either as a fixed, inevitable, and biologically determined (essentialist) or a malleable, modifiable, and flexible (nonessentialist) process (e.g., MacNeil, 2025; Weiss and Weiss, 2016), fall in the generalized VoA category. Instead, how old individuals feel compared to their chronological age [i.e., subjective or felt age (FA; Montepare, 2009)]; individuals' personal experiences as they grow older (i.e., self-perceptions of aging; e.g. Barker et al., 2007); cognitive and affective evaluations of one's own aging process [i.e., attitudes toward own aging (ATOA; e.g. Lawton, 1975; Diehl et al., 2014)]; and individuals' awareness that their behavior, performance level, or ways of experiencing life across domains have changed as a consequence of growing older [i.e., awareness of age-related change (AARC; Diehl and Wahl, 2010)] represent personal dimensions of VoA. Generalized and personal VoA have been linked to a wide range of physical and mental health, psychological well-being, quality of life, and longevity outcomes (e.g., Carbone et al., 2026; Sabatini et al., 2021; Sella et al., 2025a; Westerhof et al., 2023).

Because age-related changes in cognitive functioning typically emerge from midlife onwards (Borella et al., 2008; Craik and Salthouse, 2011) and represent major sources of concern in older age (e.g., Hertzog et al., 2008), interest has increased in the influence of VoA on cognitive functioning (e.g., Fernández-Ballbé et al., 2023, for a review). Studies on generalized VoA show that more negative beliefs concerning age and aging are associated with poorer objective cognition, particularly memory performance (Levy, 1996), and, at the long term, increased risk of cognitive decline or dementia (Levy et al., 2018). Moreover, essentialist beliefs about aging seem to negatively affect memory performance, although evidence is still limited (Weiss, 2018; Weiss and Weiss, 2016). Studies on the influence of personal VoA also indicate associations with objective cognition, though patterns differ across VoA constructs considered (Carbone et al., 2025).

A youthful FA and positive ATOA have been associated with better objective global cognitive performance (Alonso Debreczeni and Bailey, 2021; Carbone et al., 2025; Fernández-Ballbé et al., 2023), as well as better performance in memory and executive functioning domains (Fernández-Ballbé et al., 2023), although these are weak/small associations. More consistent associations have also been shown between AARC losses and poorer cognitive performance (global cognitive functioning, verbal reasoning, and short-term memory) compared to the protective role of AARC gains (Carbone et al., 2025; Sabatini et al., 2020, 2021, 2023; Voelkner and Caskie, 2023), and although the relationships are small, AARC losses emerged as stronger than AARC gains. However, despite such a promising role of VoA measures—whether generalized (e.g., old age stereotypes or essentialist beliefs about aging) or personal (e.g., FA, ATOA, or AARC)—with cognitive functioning (Fernández-Ballbé et al., 2023), as shown by a recent systematic review, none of the VoA instruments to date specifically target views of age-related cognitive changes (Sella et al., 2025a). This lack of attention to the content of perceptions and beliefs about cognitive changes with aging in VoA, in relation to cognition, is surprising, also in light of age-related cognitive changes with aging reported by older adults, particularly regarding memory functioning (Hertzog et al., 2008; Hertzog and Pearman, 2014). Older adults often evaluate their cognitive functioning more negatively than objective performance would indicate, as increased subjective cognitive complaints show (Chapman et al., 2022; Jenkins et al., 2021), a pattern now also observed in relation to VoA (e.g., Hill et al., 2025).

To date, current VoA instruments focus on global evaluations of the aging process or domains of functioning (Sella et al., 2025b, for a review). Only partially or very generally -with a single item-, they address the content of perceptions and beliefs about cognitive changes with aging.

Therefore, we aimed to develop and validate the Views of Cognitive Aging (VoCA) questionnaire, a novel instrument designed to specifically address perceptions and beliefs about age-related cognitive changes reflecting generalized and personal VoA as an integrated aspect of the broader VoA framework. The VoCA was conceived with two sections: one to capture generalized views (i.e., perceptions and beliefs about cognitive aging as it occurs in people in general) and the other to capture personal views (i.e., perceptions and beliefs about one's own cognitive aging and perceived controllability) of age-related cognitive changes occurring with aging. Items in each section were therefore formulated to represent essentialist (fixed, inevitable) and nonessentialist (malleable, modifiable) views of cognitive aging. This distinction was adapted from the (Non)Essentialist Beliefs about Aging scale ([N]EBA; Weiss and Diehl, 2021). Specifically, [N]EBA assesses essentialist and nonessentialist beliefs, that is, the extent to which the aging process is perceived as fixed and inevitable versus malleable and modifiable. It captures a core generalized dimension of VoA that goes beyond simple positive or negative evaluations and experiences of aging (i.e., how people feel about their age, assess their aging process, and interpret age-related gains and losses). We applied it specifically to cognitive aging to examine perceptions and beliefs about changes in cognitive domain-matched mechanisms (e.g., memory, attention, processing speed) known to influence complex cognitive abilities, everyday functioning (Borella et al., 2011, 2017; Wurm et al., 2017) and successful aging (Fleck et al., 2024). Thus, the VoCA goes beyond assessing perceptions of general aging processes to target perceptions and beliefs -both at the generalized and personal levels- about age-related cognitive changes.

We evaluated the two VoCA sections' psychometric properties by examining their factorial structure, internal consistency, and convergent and divergent validity through associations with well-established generalized and personal VoA constructs. Generalized VoA was assessed using the (N)EBA scale (Weiss and Diehl, 2021). Personal VoA was assessed using the FA (Lawton, 1975), ATOA (Lawton, 1975; Diehl et al., 2014), AARC-50 (Gains and Losses subscales; Carbone et al., 2024; Brothers et al., 2019), and Aging Perceptions Questionnaire (APQ; Barker et al., 2007).

A corollary aim was to examine whether VoCA dimensions were associated with objective cognitive functioning. Because the VoCA is intended to capture views of age-related cognitive changes with aging, the associations between this new questionnaire and cognition were examined by focusing on those cognitive processes that are most sensitive to age-related changes (see Craik and Salthouse, 2011) and relevant to everyday functioning (Borella et al., 2011, 2017). Therefore, working memory, attention, and processing speed tests, rather than global cognitive scores or short-term and episodic memory tests, as typically examined in the VoA literature (e.g., composite indices of overall cognitive functioning; Alonso Debreczeni and Bailey, 2021; episodic or short-term memory performance; Alonso Debreczeni and Bailey, 2021; Levy, 1996; Sabatini et al., 2023), were administered. Based on previous evidence of weak/small associations between VoA and cognition (see Alonso Debreczeni and Bailey, 2021; Carbone et al., 2025; Fernández-Ballbé et al., 2023), we expected the associations between VoCA measures and cognitive performance to be nuanced and small in magnitude.

In line with previous literature on generalized VoA (i.e., essentialism of aging; Chapman et al., 2022; Weiss and Diehl, 2021) and on multidimensional personal VoA (i.e., AARC; Brothers et al., 2017; Diehl et al., 2014), we hypothesized a two-factor structure (essentialist vs. nonessentialist views of cognitive aging) in the generalized and personal VoCA sections.

Regarding convergent validity, we expected that generalized and personal essentialist views of cognitive aging would correlate, at least modestly and positively with generalized essentialist VoA (EBA) and negative personal VoA (e.g., older FA, AARC-losses, and negative APQ dimensions) and negatively with nonessentialist VoA measures (NEBA) and positive/adaptive personal VoA (e.g., youthful FA, higher ATOA, positive APQ dimensions, and AARC-gains). Conversely, generalized and personal nonessentialist views of cognitive aging were expected to correlate positively with nonessentialist VoA (NEBA) and with positive personal VoA (youthful FA, higher ATOA, AARC-gains, higher positive APQ dimensions; Diehl et al., 2014; Sabatini et al., 2023; Tully-Wilson et al., 2021).

## 2 Method

### 2.1 Participants

A total of 727 community-dwelling middle-aged and older adults (63% women) between 50 and 84 years of age participated in the study (Table 1). All the participants were native Italian speakers recruited through word-of-mouth dissemination, informal networks, and acquaintances. Eligibility criteria included a good cognitive functioning, based on a Mini-Mental State Examination (Frisoni et al., 1993) score above the cutoff of 27; an absence of depressive symptoms, based on the Geriatric Depression Scale-15 (Sheikh and Yesavage, 2014) with the cutoff of ≤ 5; and good physical and mental health status, assessed with a demographic questionnaire (De Beni et al., 2008). The local Ethical Committee for Psychological Research approved the study.

**Table 1:** Descriptive statistics of participants' sociodemographic characteristics and measures of interest.

Variable	Statistic
Total sample	*N =* 727
Age	*M =* 63.51, *SD =* 8.63
Women, N (%)	*N =* 459 (63%)
Education	*M =* 11.76, *SD =* 4.08
MMSE (*n* = 452)	*M =* 29.28, *SD =*1.05
GDS-15	*M =* 1.60, *SD =*1.47
VoCA	
VoCA-GnE	*M =* 11.21, *SD =* 2.74
VoCA-GE	*M =* 14.88, *SD =* 3.23
VoCA-PnE	*M =* 12.83, *SD =* 4.92
VoCA-PE	*M =* 12.32, *SD =* 2.06
Generalized VoA	
EBA	*M =* 16.86, *SD =* 5.12
NEBA	*M =* 22.08, *SD =* 5.31
Personal VoA	
FA	*M =* −0.14, *SD =*0.15
ATOA	*M =* 12.44, *SD =* 1.33
AARC, Gains	*M =* 83.76, *SD =* 16.68
AARC, Losses	*M =* 50.01, *SD =* 13.73
APQ, timeline (chronic)	*M =* 3.11, *SD =* 13.95
APQ, timeline (cyclical)	*M =* 2.57, *SD =* 0.83
APQ, emotional representations	*M =* 2.53, *SD =* 0.85
APQ, control positive	*M =* 3.98, *SD =* 0.60
APQ, control negative	*M =* 3.16, *SD =* 0.53
APQ, consequences positive	*M =* 3.87, *SD =* 0.70
APQ, consequences negative	*M =* 2.89, *SD =* 0.84
Cognitive measures	
BDS—items recalled	*M =* 6.32, *SD =* 2.00
D2—number of correct items	*M =* 109.62, *SD =* 38.20
D2—total errors	*M =* 3.87, *SD =* 7.42
D2—concentration performance	*M =* 79.48, *SD =* 56.12
PCT—seconds	*M =* 136.79, *SD =* 24.94

VoCA, Views of Cognitive Aging; VoCA-GnE, Generalized Nonessentialism of Cognitive Aging; VoCA-GE, Generalized Essentialism of Cognitive Aging; VoCA-PnE, Personal Nonessentialism of Own Cognitive Aging; VoCA-PE, Personal Essentialism of Own Cognitive Aging; EBA, Essentialist Beliefs about Aging; NEBA, (N)Essentialist Beliefs about Aging; FA, Felt Age, ATOA, Attitudes Towards Own Aging; AARC, Awareness of Age-Related Change; APQ, Aging Perceptions Questionnaire; BDS, Bacward Digit Span; PCT, Pattern Comparison Task.

### 2.2 Materials

#### 2.2.1 Demographic questionnaire

The demographic questionnaire included items regarding age, education, gender, and nationality (De Beni et al., 2008). Each participant was also asked to rate their physical and psychological health on a 5-point Likert scale (1 = *very poor*, 5 = *very good*) with two *ad hoc* questions derived from the World Health Organization Quality of Life assessment (Skevington et al., 2004): “How do you rate your overall physical health?” and “How do you rate your overall psychological health?”.

#### 2.2.2 The VoCA questionnaire

The VoCA was developed to assess individuals' general and personal essentialist and nonessentialist beliefs about age-related cognitive changes, grounded in the “essentialism vs. nonessentialism of aging” framework (Weiss and Diehl, 2021; see Supplementary material, PART A for a full description of its development). The questionnaire consists of 15 items divided into two main sections. The first section (generalized VoCA; VoCA-G) was developed to measure *generalized essentialism and nonessentialism of cognitive aging*, referring to (a) the belief that cognitive aging is malleable and can be influenced by life experiences (e.g., “*With aging, memory can be improved by staying actively engaged*”) and (b) the belief that cognitive aging is fixed and immutable (e.g., “*Memory ability worsens with aging*”; Supplementary Material, PART C).

The second section (personal VoCA; VoCA-P) focuses on individuals' *personal experiences of cognitive aging*, also divided into two domains: (a) the belief that one's own cognitive aging is malleable and shaped by personal experiences, implying a sense of agency over cognitive changes (e.g., “*When I make an effort, I notice that I am able to use my mental abilities—memory, concentration, etc.—despite getting older*”) and (b) the belief that one's cognitive aging is fixed and beyond personal control, reflecting a lack of agency over cognitive changes (e.g., “*Getting older does not allow me to improve my mental abilities—memory, concentration, etc*.”).

The four dependent variables are the total scores for each of the four domains: for the first section, generalized nonessentialism and generalized essentialism of cognitive aging and for the second section, personal nonessentialism and personal essentialism of cognitive aging.

#### 2.2.3 Generalized VoA

*(Non)essentialist Beliefs about Aging ([N]EBA)*. The (N)EBA (adapted from Weiss and Weiss, 2016) is a 10-item questionnaire assessing beliefs about aging as either fixed (e.g., “Aging is solely caused by genetic factors”) or modifiable (e.g., “Age is just a number and does not say much about a person”), rated on a 6-point Likert scale (1 = *do not agree* to 6 = *absolutely agree*). Internal consistency was good for NEBA and EBA (ω = 0.76 and 0.78, respectively). Higher scores reflect stronger essentialist or nonessentialist beliefs.

#### 2.2.4 Personal VoA

*Felt Age (FA)*. Single item: “Please indicate the age that you feel, from 0 to 120 years.” Discrepancy scores were calculated as (subjective age – chronological age) / chronological age (Alonso Debreczeni and Bailey, 2021). Higher scores indicate feeling younger relative to chronological age.

*Attitudes Towards Own Aging (ATOA)*. The ATOA (taken from the Philadelphia Geriatric Center Morale Scale; Lawton, 1975) is a 5-item questionnaire (e.g., “Things keep getting worse as I get older”) with a 4-point Likert scale (1 = *strongly disagree* to 4 = *strongly agree*). Internal consistency was good (ω = 0.72). Lower scores indicate more negative ATOA; higher scores indicate more positive ATOA.

*Awareness of Age-Related Change (AARC)*. Adapted from (Brothers et al. 2019) (see also Carbone et al., 2024), this 50-item measure includes two subscales: AARC-gains (25 items, e.g., “I enjoy many things more intensively”) and AARC-losses (25 items, e.g., “Learning new things takes more time and effort”). Items are rated on a 5-point Likert scale (1 = *not at all* to 5 = *very much*) across five domains (health/physical functioning, cognitive functioning, interpersonal relationships, sociocognitive/socioemotional functioning, and lifestyle engagement). Internal consistency was good (ω = 0.92 for AARC-gains, 0.91 for AARC-losses). Higher scores reflect greater perceived gains or losses.

*Aging Perceptions Questionnaire (APQ)*. The APQ (adapted from Barker et al., 2007) consists of 32 items divided into seven subscales/dimensions: timeline chronic, timeline cyclical, emotional representations, positive control, negative control, positive consequences, and negative consequences. In this study, the internal consistency was good: timeline chronic (ω = 0.80), timeline cyclical (ω = 0.80), emotional representations (ω = 0.83), positive control (ω = 0.80), negative control (ω = 0.63), positive consequences (ω = 0.69), and negative consequences (ω = 0.85). Higher scores on each subscale indicate stronger self-perceptions of aging in each dimension.

#### 2.2.5 Cognitive measures

*Backward digit span (BDS)*. This working memory measure (Borella et al., 2008) involves presenting series of digits (one per second), and participants are required to repeat each series backward. The series start with two digits and increase to eight, with two sequences at each length. The task ends when the participant fails to correctly recall both sequences of the same length. One point is assigned for each sequence correctly recalled. The dependent variable is the total number of correctly recalled sequences.

*D2-R test*. This selective attention and concentration test is a paper-and-pencil task (Brickenkamp et al., 2013). Participants are required to scan rows of letters (targets and distractors) and mark only the target stimuli within 20 s for each row. The dependent variables are the number of correct items (number of target stimuli correctly marked), errors (number of incorrect responses), and concentration performance (correct responses minus errors and omissions). Higher scores on correct items and concentration performance indicate better selective attention and concentration whereas higher errors indicate poorer performance.

*Pattern comparison task (PCT)*. This processing speed measure is a paper-and-pencil pattern comparison test (see Borella et al., 2011). Participants are asked to complete pairs of patterns and strings of letters across two pages. The mean completion time (in seconds) is calculated for each test and serves as the dependent variable.

### 2.4 Procedure

Each participant took part in two sessions (60 min each) 1 week apart, following the same assessment order across participants. In the first session, after they completed the formal consent, the following questionnaires and tasks were administered: MMSE (only for >64), VoCA, D2-R Test, FA, PCT, APQ, and GDS-15. In the second session, participants completed the NEBA, psychological and physical health questions, AARC-50, BDS, and ATOA. This study was part of a larger project including other measures beyond the scope of this paper and therefore not considered here.

### 2.5 Statistical analyses

First, the theoretically assumed structure of the VoCA questionnaire was examined. The dataset was randomly split into two independent subsamples, with 50% assigned to the calibration subsample (*N* = 363) for exploratory factor analysis (EFA) and 50% to the validation subsample (*N* = 364) for confirmatory factor analysis (CFA). Therefore, EFA was conducted first to empirically examine whether the expected structure emerged from the selected item pool and to identify potentially problematic items, including those with weak or complex loading patterns (e.g., Goretzko et al., 2021). An oblique promax rotation was applied, and the number of factors to extract was determined based on eigenvalues greater than 1.0 and the screen test (Tabachnick and Fidell, 2007). Then, a CFA was conducted in the validation subsample to test the factor solution resulting from the EFA. Maximum likelihood estimation was used, treating response items as continuous variables, consistent with common practice in applied CFA (e.g., Rhemtulla et al., 2012) and in line with previous validation studies on subjective VoA measures (Sella et al., 2025a, for a review). Model fit was evaluated using the following indices: comparative fit index (CFI) and Tucker–Lewis index (TLI) ≥ 0.90, root mean square error of approximation (RMSEA) ≤ 0.08, and standardized root mean square residual (SRMR) ≤ 0.05 (Byrne, 2012). The sample was randomly split using *R* base functions (sample(), nrow()), and factor analyses were conducted using the psych package (Revelle, 2025).

Reliability, internal consistency (omega coefficients; ω), and item–total correlations (ITC) were calculated for each VoCA factor. Convergent validity was examined using the entire sample (*N* = 727) using Pearson's *r* correlations between VoCA and other subjective VoA measures [(N)EBA, FA, ATOA, AARC-50, and APQ]. In addition, correlations between VoCA domains and cognitive measures (BDS, D2-R Test, and PCT) were explored. For all correlations, Fisher's *r*-to-*Z* transformation (Lee and Preacher, 2013) was applied to test whether correlations among VoCA domains differed significantly while accounting for dependence between measures. Given the number of correlations tested, *p*-values were adjusted for multiple comparisons using the Benjamini-Hochberg false discovery rate procedure (Benjamini and Hochberg, 1995). All analyses were conducted in the R environment (R Core Team, 2025).

## 3 Results

The two randomly split subsamples did not differ in age, gender, education, or physical and psychological health (Supplementary Material, PART B).

### 3.1 . Exploratory factor analysis

For the first section of the VoCA (*N* = 9 items), the EFA revealed a two-factor solution explaining 36.82% of the total variance (19.14% for Factor 1, 17.69% for Factor 2). A minimum factor loading threshold of 0.40 was applied; items 6 and 9, which did not meet this criterion, were excluded. Items 1, 2, 3, and 8—reflecting malleable views of age-related cognitive changes—loaded on the first factor, interpreted as “Generalized Nonessentialist Views of Cognitive Aging” (VoCA-GnE). Items 4, 5, and 7–reflecting fixed and immutable views of cognitive aging—loaded on the second factor, interpreted as “Generalized Essentialist Views of Cognitive Aging” (VoCA-GE). Factor loadings ranged from 0.472 to 0.783. The two factors were weakly and negatively correlated (*r* = −0.20), indicating partial independence and supporting the use of oblique rotation. Comparable results emerged with orthogonal rotation, confirming the factor structure's robustness.

For the second section of the VoCA (*N* = 11 items), the EFA revealed a two-factor structure explaining 39.99% of the total variance (28.61% for Factor 1, 11.37% for Factor 2). Applying the 0.40 loading threshold led to the exclusion of item 14. Items 11, 12, 15, 17, 18, 19, and 20—capturing fixed and resigned beliefs toward one's own cognitive aging—loaded on the first factor, interpreted as “Personal Essentialist Views of Cognitive Aging” (VoCA-PE). Items 10, 13, and 16—reflecting malleable and flexible beliefs—loaded on the second factor, interpreted as “Personal Nonessentialist Views of Cognitive Aging” (VoCA-PnE). Factor loadings ranged from 0.409 to 0.877. The two factors were moderately correlated (*r* = −0.40), confirming the oblique rotation's adequacy.

### 3.2 Confirmatory factor analysis

**Figure 1:**
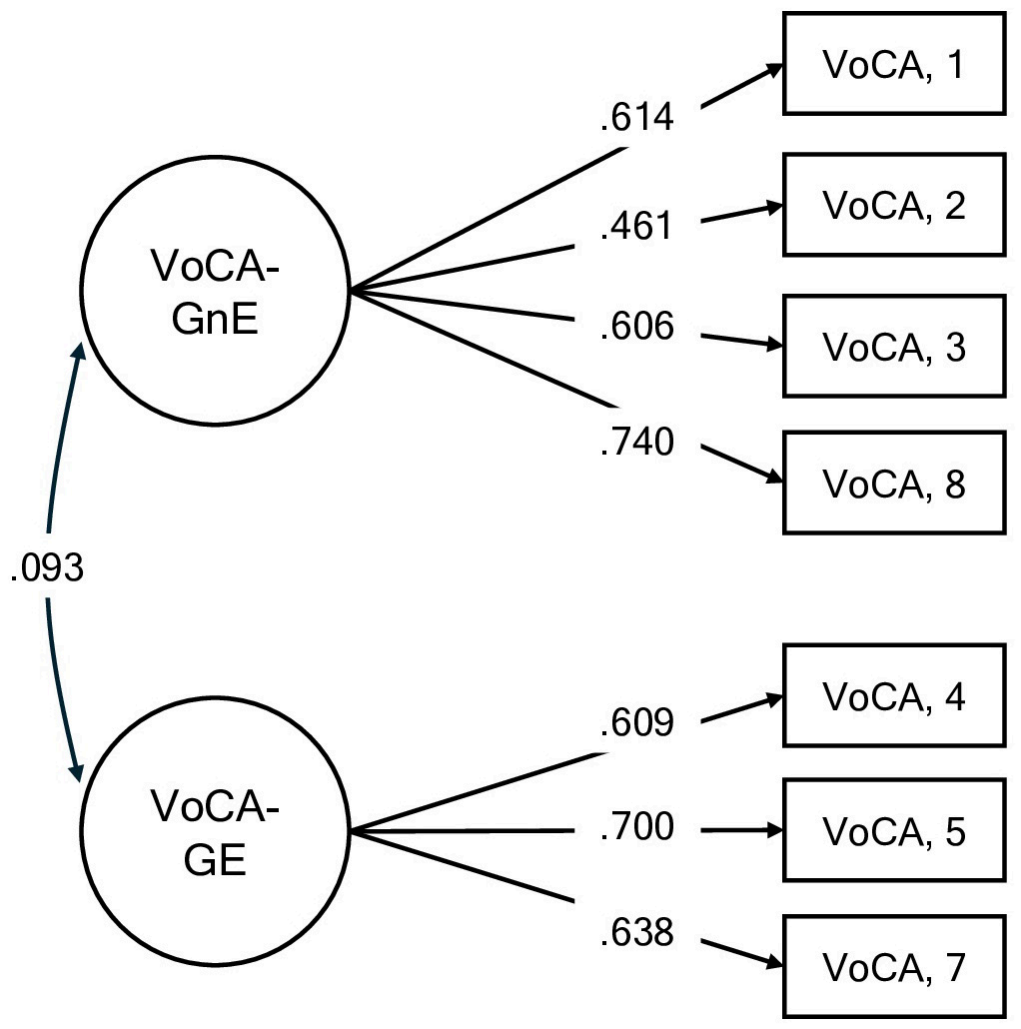
Generalized VoCA. *Note*. Standardized factor loading estimates (shown on the directional paths) from each factor to the items confirmed by the CFA. VoCA, Views of Cognitive Aging; VoCA-GnE, Generalized Nonessentialism of Cognitive Aging; VoCA-GE, generalized essentialism of cognitive aging.

For the first section of the VoCA–VoCA-G–, the two-factor model demonstrated good fit indices, χ^2^(13) = 36.84, *p* < 0.001, RMSEA = 0.07 (90% CI [0.045, 0.098]), SRMR = 0.06, CFI = 0.95, TLI = 0.91. Standardized loadings ranged from 0.461 to 0.740. The two latent dimensions were weakly and positively correlated (*r* = 0.09) (see Figure 1). The average *R*^2^ values were 37.62% for the first factor (“VoCA-GnE”) and 42.28% for the second (“VoCA-GE”; Supplementary Table B2).

**Figure 2:**
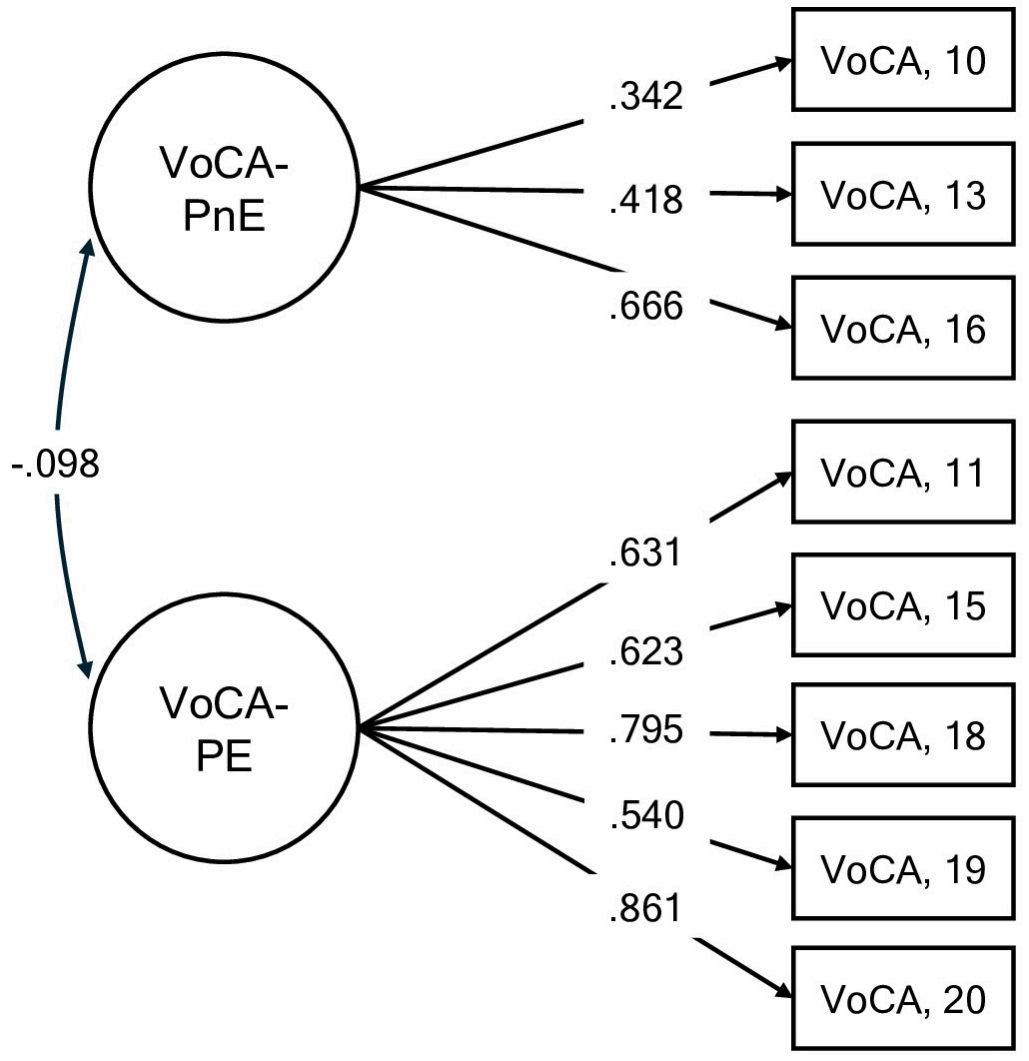
*Personal VoCA. Note*. Standardized factor loading estimates (shown on the directional paths) from each factor to the items confirmed by the CFA. VoCA, Views of Cognitive Aging; VoCA-PnE, Personal Nonessentialism of Own Cognitive Aging; VoCA-PE, Personal Essentialism of Own Cognitive Aging.

For the second section of the VoCA –VoCA-P–, the two-factor model initially did not exhibit a satisfactory fit, χ^2^(34) = 206.14, *p* < 0.001, RMSEA = 0.12, SRMR = 0.08, CFI = 0.82, TLI = 0.77. Modification indices suggested a strong correlation between items 12 and 17 (MI = 72.27, EPC = 0.49), suggesting redundancy. After these two items were removed, the model showed improved fit indices, χ^2^(19) = 59.03, *p* < 0.001, RMSEA = 0.08, SRMR = 0.04, CFI = 0.94, TLI = 0.92. Standardized loadings ranged from 0.342 to 0.861. The two latent factors were weakly and negatively correlated (*r* = −0.10) (see Figure 2). The *R*^2^ values were 24.5% for the first factor (“VoCA-PnE”) and 49.01% for the second factor (“VoCA-PE”; Supplementary Table B3).

### 3.3 Item and subscale reliability

No item showed an ITC value below the 0.30 threshold. For the first section, ω was 0.76 for VoCA-GnE and 0.74 for VoCA-GE. For the second section, ω values were 0.60 for VoCA-PnE and 0.85 for VoCA-PE. These coefficients indicate good internal reliability (Kalkbrenner, 2023), although the VoCA-PnE subscale showed a marginally acceptable reliability.

### 3.4 Convergent validity

Supplementary Table B4 shows the correlations between the VoCA and VoA measures. For the first section, VoCA-GnE was moderately positively associated with NEBA and showed small negative correlations with EBA whereas VoCA-GE showed small to moderate correlations with NEBA and EBA. Regarding personal VoA, VoCA-GnE showed small positive correlations with AARC-gains, APQ-control positive, APQ-control negative, and APQ-consequences positive and small negative correlations with FA (feeling younger), AARC-losses, and other APQ dimensions (timeline chronic, timeline cyclical, emotional representations, consequences negative, and control negative). In contrast, VoCA-GE showed a small and positive association with FA (feeling older), a small and negative association with ATOA, a small to moderate positive correlation with AARC (gains and losses), small correlations with APQ dimensions (timeline chronic, timeline cyclic, emotional representations, consequences positive and negative), and a small and negative correlation with APQ control negative. Fisher's *z* tests indicated that VoCA-GnE was significantly more strongly related to adaptive and controllable aging beliefs (APQ-control positive, APQ-consequences positive, and AARC-gains) than VoCA-GE whereas VoCA-GE showed stronger associations with maladaptive and more deterministic perceptions (AARC-losses, APQ-consequences negative, APQ-timeline chronic and cyclical), supporting the conceptual distinction between flexible and fixed cognitive-aging beliefs.

For the personal VoCA section, VoCA-PnE showed a moderate positive correlation with NEBA and a small negative correlation with EBA whereas VoCA-PE showed a small positive correlation with EBA and was not significantly related to NEBA. As for personal VoA, VoCA-PnE showed small negative correlations with FA (feeling younger) and negative APQ dimensions (timeline chronic and cyclical, emotional representations, consequences negative, and control negative) and small to moderate positive correlations with ATOA, AARC-gains, APQ-control positive, APQ-control negative, and APQ-consequences positive. VoCA-PE showed small positive correlations with FA (feeling older), a moderate positive correlation with AARC-losses, small to moderate positive correlations with AARC-gains and APQ dimensions (timeline chronic, timeline cyclical, emotional representations, and consequences negative), and small negative correlations with APQ-control positive and APQ-control negative. No significant associations were found with ATOA or APQ-consequences positive. Fisher's *z* tests confirmed that VoCA-PnE exhibited significantly stronger associations with positive and controllable aging beliefs (AARC-gains, APQ-control positive, APQ-consequences positive) whereas VoCA-PE showed stronger correlations with negative and more deterministic aging perceptions (AARC-losses, APQ-consequences negative, APQ-timeline chronic and cyclical; Supplementary Table B4).

### 3.5 Associations between VoCA and cognitive performance

Supplementary Table B5 shows correlations between VoCA and cognitive measures. For the generalized VoCA section, VoCA-GnE showed small but significant positive correlations with BDS and with D2 correct responses and D2 concentration performance and showed negative correlations with D2 errors and PCT. In contrast, VoCA-GE did not show significant correlations with any cognitive measure. Fisher's *z* tests confirmed that the correlations of VoCA-GnE with cognitive measures were generally stronger than those of VoCA-GE, except for D2 errors and pattern comparison.

For the personal VoCA section, VoCA-PnE was positively associated with BDS, D2 correct responses, and D2 concentration performance and negatively associated with PCT. In contrast, VoCA-PE was negatively associated with BDS, D2 correct responses, and D2 concentration performance and positively associated with D2 errors while showing no significant association with PCT. Fisher's *z* tests confirmed that the correlations of VoCA-PnE with BDS, D2 correct responses, and D2 concentration performance were generally stronger than those of VoCA-PE whereas the opposite pattern emerged for D2 errors.

## 4 Discussion

This study presents a new scale to measure specifically views of cognitive aging (i.e., perceptions and beliefs about cognitive changes with aging from middle-aged to older adults): the VoCA. The development and validation of the VoCA questionnaire is rooted in essentialist and nonessentialist views of cognitive aging at the generalized and personal levels, encompassing the multidimensionality of the VoA macroconstruct (Shrira et al., 2022; Weiss and Diehl, 2021). It thus extends current VoA frameworks (Shrira et al., 2022) specifically to cognitive aging.

Our findings supported the two-factor structure for the generalized and personal VoCA sections. For the generalized VoCA section (7 items), EFA revealed two factors, with items loading as expected on nonessentialist—malleable and flexible—vs. essentialist—fixed and immutable—perceptions and beliefs about age-related cognitive changes, reflecting generalized nonessentialist (four items; VoCA-GnE) and essentialist (three items; VoCA-GE) views of cognitive aging, respectively. The subsequent CFA yielded good fit indices and reliability.

Similarly, for the personal VoCA section, factor analyses indicated two distinct factors, with items tapping nonessentialist—malleable, flexible, and controllable–views of one's own cognition versus essentialist—fixed, resigned, and uncontrollable—views of one's own cognition, capturing personal nonessentialism (VoCA-PnE) and essentialism (VoCA-PE), respectively. Although some item exclusions and model adjustments (i.e., correlated residuals for items 12 and 17) were needed, the final model showed good fit and internal consistency.

The weak correlations between nonessentialist and essentialist factors in each VoCA section suggest even greater independence than observed in established VoA measures (e.g., NEBA-EBA for general VoA, AARC gains-losses for personal VoA; Brothers et al., 2019; Weiss and Diehl, 2021). Such a result indicates that the VoCA dimensions are sufficiently distinct to independently measure essentialist and nonessentialist views of cognitive aging. The two-factor structure of VoCA for generalized and personal sections extends the operationalization of VoA constructs, distinguishing generalized vs. personal (self) views (Shrira et al., 2022) and essentialist vs. nonessentialist beliefs (Weiss and Diehl, 2021) about aging, to the specific domain of cognitive aging.

The convergent and divergent validity findings demonstrated that the VoCA questionnaire assesses a distinct yet related VoA construct compared to the ones depicted by the considered existing—and classical—VoA instruments.

Generalized and personal nonessentialist views about cognitive aging (VoCA-GnE, VoCA-PnE), as expected, were positively associated with NEBA for generalized VoA and with adaptive/positive personal VoA [i.e., youthful FA, higher ATOA (only VoCA-PnE), greater AARC-gains, and more positive APQ dimensions (e.g., positive control, positive consequences)]. At the same time, VoCA nonessentialism, as expected, was negatively related to maladaptive/negative personal VoA [i.e., older FA, higher AARC-losses, and negative APQ dimensions (timeline chronic/cyclical, emotional representations, negative control/consequences)]. In contrast, generalized and personal essentialist views of cognitive aging (VoCA-GE, VoCA-PE) showed opposite patterns, generalized essentialist views of cognitive aging related positively to EBA, older FA, lower ATOA (only for VoCA-GE), and increased AARC-losses and negative APQ dimensions, but they had weak or nonsignificant correlations with personal VoA, in line with our expectations. These patterns support previous conceptualizations of essentialist beliefs about aging (Weiss, 2018; Weiss and Weiss, 2016) as reflecting negative age stereotypes and some reduced perceived cognitive malleability whereas nonessentialist beliefs may foster more flexible and agentic views of cognitive aging.

Notably, VoCA also showed differentiated but modest associations with some of the core cognitive mechanisms in aging considered here to assess objective cognitive performance. Generalized and personal nonessentialist views of cognitive aging (VoCA-GnE, VoCA-PnE) were modestly but consistently associated with better working memory (higher BDS performance), selective attention and concentration (D2 correct responses and concentration performance), and faster processing speed (PCT). These results extend previous evidence linking VoA constructs to some core cognitive domains sensitive to aging (Carbone et al., 2025; Fernández-Ballbé et al., 2023; Levy et al., 2018; Sabatini et al., 2023). Like broader VoA measures-where personal measures typically correlate more strongly than generalized ones (e.g., Fernández-Ballbé et al., 2023)-VoCA showed similar modest associations with objective cognitive performance. Nonetheless, and interestingly, VoCA extends VoA associations with cognition beyond global evaluations of aging process and valenced assessments (e.g., perceived gains and losses) to fixedness and malleability of views of cognitive aging trajectories across both generalized and personal VoCA sections.

Personal essentialist views of cognitive aging (VoCA-PE) were modestly related to poorer working memory performance, an increase in attentional errors (higher D2 errors), and lower concentration performance, but not to the speed at which information is processed. These correlations suggest that personal nonessentialist perceptions and beliefs about one's own cognitive aging may be particularly relevant for tasks that are more demanding in terms of required cognitive control, such as tasks involving working memory and selective attention, and that are experienced as more complex than processing speed tasks, which may appear easier to perform. In contrast, generalized essentialist views of cognitive aging (VoCA-GE) showed no significant associations with any of the cognitive domains considered here, which may reflect that generalized beliefs about cognitive aging are more strongly rooted in socially shared age stereotypes (e.g., Levy et al., 2012, 2018) than in individuals' own cognitive experiences. Such a different pattern of correlations between personal and general essentialist VoCA seems in accordance with VoA models that emphasize the specific role of self-perceptions of aging for behavior and health-related outcomes (Diehl et al., 2014; Shrira et al., 2022).

Despite such interesting findings, some limitations should be acknowledged. First, our sample consisted of cognitively healthy, community-dwelling middle-aged and older adults (50–84 years), representing typical demographic distributions for this age range, though this restricts generalisability to individuals with more heterogeneous cognitive profiles (e.g., people with -subjective- cognitive decline). Second, women were overrepresented here (63% of the total sample). Measurement invariance across gender was supported for the personal VoCA section, indicating that its two-factor structure was comparable across men and women. For the generalized VoCA section, fit indices remained stable across models, including the scalar model, although the chi-square difference test between the configural and metric models was significant (see Supplementary Table B6). This finding may suggest some gender-related differences in the equivalence of factor loadings for generalized beliefs about cognitive aging, rather than differences in the overall two-factor structure of the VoCA-G. Gender differences in aging-related cognitive trajectories (e.g., Ferretti et al., 2018; Sundermann et al., 2017) and the impact of gender stereotypes on health and aging-related expectations (e.g., Brown, 2015; Levy, 2009) may have influenced perceptions and beliefs about cognitive aging and cognitive performance. Therefore, there is the need for more balanced sex and gender representation to deepen the influence of such individual characteristics in future research using the VoCA and, more generally, in VoA studies. Third, although the overall validity of this new instrument was generally good, the internal consistency of the VoCA-PnE subscale was marginally acceptable. This may partly reflect the small number of items included in the VoCA-PnE, as omega estimates are influenced by the number of items (e.g., Edwards et al., 2021), as well as by the heterogeneity of the retained item content. In line with previous studies (Weiss and Weiss, 2016; Weiss and Diehl, 2021), nonessentialist views of aging can be understood as referring to aging as a relatively malleable and modifiable process. In the present study, the retained VoCA-PnE items appear to capture different aspects of these views, including understanding one's cognitive functioning with aging, perceiving the benefits of effort when using cognitive abilities, and beliefs about maintaining good memory and cognitive skills through practice. By contrast, essentialist views of aging emphasize aging as a relatively fixed, inevitable, and uncontrollable process, which may reflect a more homogeneous core belief system and help explain the stronger internal consistency and higher explained variance of the VoCA-PE subscale (49%) compared with VoCA-PnE (25%). Fourth, because we conducted a cross-sectional validation study –typically used in VoA scale validations (e.g., Brothers et al., 2019; Sella et al., 2025b for a review; Weiss and Diehl, 2021)—with a fixed task presentation order, our findings would need a longitudinal replication to test the predictive validity of VoCA over time.

Nonetheless, the convergent and divergent validities, along with the correlations with cognitive measures selected, make the VoCA a promising questionnaire for applied implications. On the one hand, the VoCA may help identify individuals who endorse strongly essentialist views of cognitive aging at the generalized and personal levels. These individuals may be at greater risk for fixed and immutable views of everyday cognitive performance, heightened worry or complaints about cognitive decline, and withdrawal from cognitively demanding activities, which are known to support and foster cognitive functioning in adulthood and older age (e.g., Borella et al., 2023). On the other hand, a stronger nonessentialist view of changes with cognitive aging may indicate a more malleable, growth- and agency-oriented perspective that could be leveraged in preventive cognitive and metacognitive interventions (e.g., Sella et al., 2023). Integrating the VoCA into screening protocols and longitudinal studies may clarify whether shifts toward more nonessentialist views prospectively predict more favorable cognitive trajectories beyond traditional risk factors (Livingston et al., 2024). Indeed, whereas most modifiable risk factors for cognitive decline are objectively measured (e.g., lifestyle, health conditions), much less is known about how individuals perceive and interpret age-related cognitive changes and how these beliefs contribute to risk for cognitive decline. Furthermore, using the VoCA to tailor psychoeducational or cognitive training programs—for instance, by challenging fixed/deterministic views, highlighting evidence for plasticity, and reinforcing a sense of agency over cognitive functioning—may enhance engagement and the effectiveness of interventions aimed at maintaining cognitive health from midlife onward.

To conclude, the VoCA, a distinct but interrelated new VoA measure at the generalized and personal levels, moves beyond the traditional generalized versus personal distinction in existing VoA measures by capturing not only beliefs and expectations about aging-related changes but also the essentialist–nonessentialist nature of cognitive aging (Weiss, 2018; Weiss and Diehl, 2021; Weiss et al., 2022; Weiss and Weiss, 2016). This new instrument also enriches existing VoA frameworks (Shrira et al., 2022) through its modest but significant associations with objective cognitive performance, suggesting that how people conceptualize cognitive aging—as fixed or malleable in general and as uncontrollable or agentic with respect to their own cognition—is linked to performance in age-sensitive cognitive domains. It thus opens up new avenues for research and interventions on subjective cognitive aging in midlife and older adulthood.

## Data Availability

The raw data supporting the conclusions of this article will be made available by the authors, without undue reservation.
