# Supplementary material for: Views of cognitive aging in midlife and older age: development of a new scale
Source: Front Psychol. 2026 May 20;17:1837995. doi: 10.3389/fpsyg.2026.1837995 (PMC13230079; doi:10.3389/fpsyg.2026.1837995)
Supplement: Supplementary file 1 [file Data_Sheet_1.docx]

Supplementary Material

Views of Cognitive Aging in Midlife and Older Age: Development of a New Scale

# 1. PART A – Details for the Development of the VoCA Questionnaire

# 2. PART B – Psychometric Results and Validation Analyses

# 3. PART C – VoCA Questionnaire and Scoring System

# 1. PART A - Details for the Development of the VoCA Questionnaire

The first version of the VoCA questionnaire was developed through a multistep process aimed at generating a comprehensive set of items that would reliably capture individuals’ beliefs and perceptions about age-related cognitive changes with aging. Starting from our previous systematic review (Sella et al., 2025), which summarized existing self-report VoA instruments, we identified and synthesised content specifically linked to perceptions and beliefs about age-related cognitive changes among the available VoA measures validated among older adults.

A preliminary discussion was then conducted among the authors (ES, EC, EB) together with three experts in psychology of aging and clinical psychology (MV, JCM, VV). These exchanges guided two core activities: (a) identifying items and conceptual domains from previously validated VoA instruments that were relevant for delineating the new construct of “Views of Cognitive Aging (VoCA),” embedded in the VoA framework, (b) and generating additional items to ensure that the VoCA would sufficiently/adequately cover generalized and personal VoA related to cognitive aging.

To further refine the conceptualization and wording of potential items, six focus groups were conducted with older adults (*N* = 6 or 7 per group, aged 60–84 years). To capture perceptions of and beliefs about age-related cognitive changes from individuals with varying familiarity with cognitive functioning and cognitive aging, three groups included participants without prior exposure to cognitive training whereas the remaining three involved individuals who had attended cognitive training at the Unit and Lab of Psychology of Aging (University of Padova). During these sessions, a trained moderator (ES) facilitated discussions concerning participants’ understanding of cognitive abilities and their experiences of age-related cognitive changes, prompting reflection on positive and negative aspects. Participants responded to general questions (e.g., “What do you know about cognitive abilities?”) and more targeted prompts (e.g., “Which factors can influence your mental abilities?” and “Do these factors affect how you think about your mental abilities?”). All discussions were audio-recorded and transcribed verbatim.

Two independent raters (ES and CDV, an expert psychologist in qualitative methods and focus groups) analysed the transcripts, extracting relevant statements and categorising them into two broad areas: general knowledge and beliefs about cognitive functioning in aging and personal knowledge and beliefs concerning changes in one’s own cognitive abilities. Based on these categories, an initial item pool that explicitly reflected the distinction between generalized and personal VoA (Palgi et al., 2022) was adapted to the domain of cognitive aging. This resulted in items capturing generalized and personal views of cognitive aging.

The initial pool for the VoCA consisted of 33 items. This first version was subsequently evaluated in an additional focus group of older adults (*N* = 7, aged 60–80 years), none of whom had participated in cognitive training. The goal of this final qualitative step was to assess item clarity, comprehensibility, and perceived relevance (accordingly to the COSMIN methodology; Mokkink et al., 2018; Terwee et al., 2018). Based on participants’ feedback, the item set was refined and reduced to 20 items, organized into two sections: The first section (nine items) assessed generalized views of cognitive aging (generalized VoCA), and the second section (11 items) assessed personal views of cognitive aging (personal VoCA).

The structure of both sections was inspired by the conceptual distinction between essentialist and nonessentialist beliefs about aging (Weiss & Diehl, 2020). The generalized VoCA section therefore captures beliefs about whether cognitive aging is malleable and influenced by lifestyle or environmental factors (generalized nonessentialism) or instead fixed and unchangeable (generalized essentialism). Conversely, the personal VoCA section reflects individuals’ personal interpretations/knowledge of their own cognitive aging, distinguishing between beliefs that their cognitive trajectories are shaped by their own experiences and behaviours/choices, associated with a sense of agency and controllability (personal nonessentialism), and beliefs that cognitive aging is predetermined and beyond personal control (personal essentialism).

# 2. PART B - Psychometric Results and Validation Analyses

# 2.1 Psychometric Results and Validation Analyses

**Table B1.** *Comparison of the Two Subsamples.*

|  | EFA  (N= 363) | | CFA  (N= 364) | |  |  |  |
| --- | --- | --- | --- | --- | --- | --- | --- |
|  | M | SD | M | SD | *t* | *p* | *Cohen’s d* |
| Age | 63.74 | 8.78 | 63.29 | 8.47 | -.699 | .485 | -.052 |
| Gender° (%, female) | 61% |  | 62% |  | 1.583 | .208 |  |
| Education | 11.66 | 4.29 | 11.86 | 3.86 | .675 | .500 | .050 |
| MMSE* | 29.30 | 1.05 | 29.28 | 1.04 | .064 | .949 | .005 |
| Physical health | 2.89 | .60 | 2.89 | .63 | -.904 | .366 | -.067 |
| Psychological health | 4.02 | .62 | 3.98 | .63 | -.699 | .485 | -.052 |

*Note.* **n* = 221 for EFA, 238 for CFA; °*X*^2^ test (1 = male, 2 = female).

**Table B2.** *CFA Results for Generalized VoCA: Standardized Factor Loadings (*λ*), Item–Total correlations (ITC), Reliability (*ω*), and Variance Explained (*R*^2^).*

|  |  | **Factor 1 –**  **VoCA-GnE** | **Factor 2 –**  **VoCA-GE** |
| --- | --- | --- | --- |
| Item | *R*^2^ | *λ* | *λ* |
| 1. As we age/with aging, memory can be improved by staying actively engaged. | .377 | .614 |  |
| 2. Age is just a number and says little about a person’s mental abilities. | .461 | .461 |  |
| 3. The aging of mental abilities (memory, concentration, etc.) can be controlled and slowed down. | .606 | .606 |  |
| 8. Using and keeping mental abilities (memory, concentration, etc.) always active can influence how they change with aging. | .740 | .740 |  |
| 4. With aging, feeling that mental abilities (memory, concentration, etc.) function more slowly is normal. | .609 |  | .609 |
| 5. Memory ability worsens with aging. | .700 |  | .700 |
| 7. It is normal for mental abilities (memory, concentration, etc.) to decline with aging. | .638 |  | .638 |
| *ω* |  | .76 | .74 |
| ITC |  | .54–.87 | 73–84 |
| *R*^2^ (%) |  | .376 (37.62%) | .423 (42.28%) |

*Note.* VoCA= views on cognitive aging; VoCA, GnE = generalized nonessentialism of cognitive aging; VoCA, GE = general essentialism of cognitive aging. Items excluded from the EFA analysis because they did not reach the factor loading threshold of .40 and therefore omitted from the subsequent CFA: Item 6: “The way mental abilities (memory, concentration, etc.) function is genetically determined”, Item 9: “There is no way to recover memory loss that occurs with aging”.

**Table B3.** *CFA Results for Personal VoCA: Standardized Factor Loadings (*λ*), Item–Total Correlations (ITC), Reliability (*ω*), and Variance Explained (*R*^2^).*

|  |  | **Factor 1 –**  **VoCA-PnE** | **Factor 2 –**  **VoCA-PE** |
| --- | --- | --- | --- |
| Item | *R*^2^ | *λ* | *λ* |
| 10. It is important to understand —know and learn— how my mental abilities (memory, concentration, etc.) function with aging. | .117 | .342 |  |
| 13. When I make an effort, I notice that I am able to use my mental abilities (memory, concentration, etc.) despite getting older | .175 | .418 |  |
| 16. If I continue to exercise my memory, it will stay fairly good as I age. | .443 | .666 |  |
| 11. I notice that as I age, it becomes harder for me to clearly remember information in everyday situations. | .398 |  | .631 |
| 15. The slowness of my mental abilities during daily activities is due to aging. | .388 |  | .623 |
| 18. With aging, I have less control over my memory —i.e., remembering when I want to. | .632 |  | .795 |
| 19. Getting older does not allow me to improve my mental abilities (memory, concentration, etc.) | .292 |  | .540 |
| 20. With aging, I cannot use my mental abilities (memory, concentration, etc.) as well as I would like. | 742 |  | .861 |
| *ω* |  | .60 | .85 |
| ITC |  | .47-91 | .58-.93 |
| *R*^2^ (%) |  | .245 (24.49%) | .490 (49.01%) |

*Note.* VoCA= views on cognitive aging, PE = personal essentialism of cognitive aging, VoCA, PnE = personal nonessentialism of own cognitive aging, VoCA, PE = personal essentialism of own cognitive aging. Items excluded from the EFA analysis because they did not reach the factor loading threshold of .40 and were therefore omitted from the subsequent CFA: Item 12: “I cannot do anything to prevent my memory from worsening with aging”, Item 14: “Aging does not influence my attention during daily activities”, Item 17: I am aware that I cannot do anything to prevent my mental abilities (memory, concentration, etc.) from worsening”.

**Table B4.** *Convergent and Divergent Validities for Generalized and Personal VoCA.*

|  | Generalized VoCA | | *z (Fisher’s r-to-z)* | Personal VoCA | | *z (Fisher’s r-to-z)* |
| --- | --- | --- | --- | --- | --- | --- |
|  | *VoCA-GnE* | *VoCA-GE* |  | *VoCA-PnE* | *VoCA-PE* |  |
| *Generalized VoA* |  |  |  |  |  |  |
| NEBA | **.495^*^** | **-.225^*^** | **14.29^*^** | **.355^*^** | -.077 | **8.14^*^** |
| EBA | **-.381^*^** | **.216^*^** | **-11.54^*^** | **-.206^*^** | **.212^*^** | **-7.74^*^** |
| *Personal VoA* |  |  |  |  |  |  |
| Felt age | **-.158^*^** | **.127^*^** | **-5.35^*^** | **-.147^*^** | **.278^*^** | **-7.90^*^** |
| ATOA | .007 | **-.084^*^** | -1.43 | **.116^*^** | .051 | 1.18 |
| AARC, Gains | **.170^*^** | **.137^*^** | 0.62 | **.198^*^** | **.089*** | **2.01^*^** |
| AARC, Losses | **-.168^*^** | **.320^*^** | **-9.32^*^** | **-.116^*^** | **.422^*^** | **-10.27^*^** |
| APQ, timeline (chronic) | **-.171^*^** | **.186^*^** | **-6.73^*^** | **-.108^*^** | **.303^*^** | **-7.67^*^** |
| APQ, timeline (cyclical) | **-.086^*^** | **.149^*^** | **-4.40^*^** | **-.124^*^** | **.296^*^** | **-7.83^*^** |
| APQ, emotional representations | **-.151^*^** | .077 | **-4.27^*^** | **-.103^*^** | **.222^*^** | **-5.99^*^** |
| APQ, control positive | **.387^*^** | −.010 | **7.71^*^** | **.357^*^** | **-.129^*^** | **9.15^*^** |
| APQ, control negative | **.273^*^** | **−.130^*^** | **7.63^*^** | **.167^*^** | **-.215^*^** | **7.04^*^** |
| APQ, consequences positive | **.103^*^** | .049 | 1.02 | **.154^*^** | -.060 | **3.92^*^** |
| APQ, consequences negative | **-.161^*^** | **.260^*^** | **-7.97^*^** | **-.097^*^** | **.357^*^** | **-8.55^*^** |

*Note.* VoCA= views on cognitive aging, PE = personal essentialism of cognitive aging, VoCA, PnE = personal nonessentialism of own cognitive aging, VoCA, PE = personal essentialism of own cognitive aging; VoCA, GnE = generalized nonessentialism of cognitive aging, VoCA, GE = generalized essentialism of cognitive aging; EBA = essentialist beliefs about aging; NEBA = nonessentialist beliefs about aging; ATOA = attitudes towards own aging; AARC = awareness of age-related change; APQ = aging perceptions questionnaire. In bold type *p* values **p < .05; **p < .01. ***p < .001*

**Table B5.** *Correlations Among Generalized and Personal VoCA and Cognitive Domains.*

|  | Generalized VoCA | | *z (Fisher’s r-to-z)* | Personal VoCA | | *z (Fisher’s r-to-z)* |
| --- | --- | --- | --- | --- | --- | --- |
|  | *VoCA-GnE* | *VoCA-GE* |  | *VoCA-PnE* | *VoCA-PE* |  |
| BDS | **.110^*^** | -.066 | **3.320^***^** | **.106^*^** | **-.165^*^** | **4.927^***^** |
| D2, corrects | **.121^*^** | -.050 | **3.238^***^** | **.086^*^** | **-.117^*^** | **3.645^***^** |
| D2, errors | **-.092^*^** | .010 | -1.830 | -.039 | **.099^*^** | **-2.501^*^** |
| D2, concentration performance | **.094^*^** | -.041 | **2.455^*^** | **.081^*^** | **-.122^*^** | **3.641^***^** |
| Pattern comparison test | **-.130^***^** | -.035 | -1.843 | **-.094^*^** | .041 | **-2.358^*^** |

*Note:* VoCA= views on cognitive aging, PE = personal essentialism of cognitive aging, VoCA, PnE = personal nonessentialism of own cognitive aging, VoCA, PE = personal essentialism of own cognitive aging; VoCA, GnE = generalized nonessentialism of cognitive aging, VoCA, GE = generalized essentialism of cognitive aging; BDS = backward digit span. In bold type *p* values **p < .05; **p < .01, ***p < .001.*

**Table B6.** Fit Indices for Testing Measurement Invariance Across Gender for Generalized and Personal VoCA in the Full Sample.

| **Section** | **Model** | **χ²** | **df** | **CFI** | **RMSEA** | **SRMR** | **Δχ²** | **Δdf** | **p** |
| --- | --- | --- | --- | --- | --- | --- | --- | --- | --- |
| Generalized VoCA | Configural | 94.43 | 26 | .978 | .085 | .069 | — | — | — |
|  | Metric | 105.39 | 31 | .976 | .081 | .071 | 13.29 | 5 | .021 |
|  | Scalar | 114.94 | 50 | .979 | .060 | .070 | 15.94 | 19 | .661 |
| Personal VoCA | Configural | 101.18 | 38 | .990 | .068 | .061 | — | — | — |
|  | Metric | 108.85 | 44 | .989 | .064 | .063 | 7.62 | 6 | .267 |
|  | Scalar | 119.32 | 66 | .991 | .047 | .062 | 18.52 | 22 | .675 |

Note. Configural invariance (i.e., invariance of the number of factors and the pattern of factor-indicator relationships); metric invariance (i.e., invariance of the factor loadings across groups); scalar invariance (i.e., invariance of the item intercepts across groups).

# 3. PART C - VoCA Questionnaire and Scoring System

**VoCA Questionnaire and Scoring System**

The final version of the VoCA questionnaire is presented below with its two sections, the generalized VoCA (VoCA-G) section and the personal VoCA (VoCA-P) section, along with the scoring system to calculate the dimensions for each section. An English translation follows below.

***English Version***

**VoCA-G**

**Generalized - Views of Cognitive Aging**

INSTRUCTIONS: The following statements address **generalized beliefs about aging and age-related changes with aging in mental abilities**—such as memory (ability to learn/recall facts or information), attention (ability to stay focused), and processing speed—used to manage daily activities, tasks, and challenges.

Please read each statement carefully and indicate your level of agreement on the following scale:

1 = completely disagree, 2 = slightly agree, 3 = moderately agree, 4 = quite agree, 5 = strongly agree.

There are no “right” or “wrong” answers. We are interested in your opinions.

|  | Completely disagree (1) | Slightly agree (2) | Moderately agree (3) | Quite agree (4) | Strongly agree (5) |
| --- | --- | --- | --- | --- | --- |
| 1. As we age/with aging, **memory can be improved by staying actively engaged**. |  |  |  |  |  |
| 1. **Age** is just a number and **says little about a person’s mental abilities**. |  |  |  |  |  |
| 1. The aging of mental abilities —memory, concentration, etc.— can be **controlled and slowed down**. |  |  |  |  |  |
| 1. With aging, feeling that **mental abilities** —memory, concentration, etc. — function more **slowly** is normal. |  |  |  |  |  |
| 1. Memory ability **worsens** with aging. |  |  |  |  |  |
| 1. It is normal for **mental abilities** (memory, concentration, etc.) **to decline** with aging |  |  |  |  |  |
| 1. Using and keeping mental abilities —memory, concentration, etc.— always active can **influence** how they change with aging. |  |  |  |  |  |

**VoCA-P**

**Personal - Views of Cognitive Aging**

INSTRUCTIONS: The following statements address **YOUR** personal beliefs and experiences regarding aging and age-related changes in **YOUR** mental abilities —e.g., memory, attention, and processing speed.

Please read each statement carefully and indicate your level of agreement on the following scale:
1 = Completely disagree, 2 = Slightly agree, 3 = Moderately agree, 4 = Quite agree, 5 = Strongly agree.

There are no “right” or “wrong” answers. We are interested in understanding **YOUR** personal experiences of age-related changes in **YOUR** mental abilities that you may have noticed in daily life.

|  | Completely disagree (1) | Slightly agree (2) | Moderately agree (3) | Quite agree (4) | Strongly agree (5) |
| --- | --- | --- | --- | --- | --- |
| 1. It is important **to understand** —know and learn— **how my mental abilities** —memory, concentration, etc.—**function** with aging. |  |  |  |  |  |
| 1. **I notice that** as I age, it becomes **harder** **for me** **to clearly remember** information in everyday situations. |  |  |  |  |  |
| 1. When I **make an effort**, **I notice that I am still able** **use my mental** abilities —memory, concentration, etc. — despite aging. |  |  |  |  |  |
| 1. The **slowness** of **my** **mental abilities** during daily activities is due to aging. |  |  |  |  |  |
| 1. If I continue to **exercise my memory**, **it will stay** fairly good as I age. |  |  |  |  |  |
| 1. With aging, **I have less control over my memory** —i.e., remembering when I want to. |  |  |  |  |  |
| 1. Getting older **does not allow me to improve my mental abilities**—memory, concentration, etc. |  |  |  |  |  |
| 1. With aging, **I cannot use my mental abilities** —memory, concentration, etc. — as well as I would like. |  |  |  |  |  |

***Scoring system***

| VoCA-G: Generalized - Views of Cognitive Aging |  |
| --- | --- |
| VoCA-GnE = Generalized non-Essentialism of cognitive aging | SUM: item 1, 2, 3, 7 |
| VoCA-GE = Generalized Essentialism of cognitive aging | SUM: item 4, 5, 6 |
| VoCA-P: Personal - Views of Cognitive Aging |  |
| VoCA-PnE = Personal non-Essentialism of own cognitive aging | SUM: item 8, 10, 12 |
| VoCA-PE = Personal Essentialism of own cognitive aging | SUM: item 9, 11, 13, 14, 15 |
